# Molecular Profiling: Genomic-Guided Therapy for Lung Adenocarcinoma

**DOI:** 10.7759/cureus.84789

**Published:** 2025-05-25

**Authors:** Hector A Nieves Figueroa, William Rodriguez Cintron

**Affiliations:** 1 Department of Pulmonary and Critical Care Medicine, Veterans Affairs (VA) Caribbean Healthcare System, San Juan, PRI; 2 Department of Pulmonary and Critical Care Medicine, Veterans Affairs (VA) Caribbean Healthcare Systems, San Juan, PRI

**Keywords:** capmatinib therapy, lung adenocarcinoma, met exon 14, molecular profiling, targeted therapy

## Abstract

Non-small cell lung cancer (NSCLC) accounts for the majority of lung cancer cases and is frequently diagnosed at advanced stages, leading to poor survival outcomes. Molecular analysis can identify actionable mutations, such as mesenchymal-epithelial transition (MET) exon 14 skipping mutations, which serve as therapeutic targets. Capmatinib, a selective MET tyrosine kinase inhibitor (TKI), has emerged as a promising targeted therapy for this molecular subtype. We present the case of an 83-year-old former smoker with a history of chronic obstructive pulmonary disease who was diagnosed with stage 4A metastatic lung adenocarcinoma. Molecular profiling revealed a MET exon 14 skipping mutation. The patient was initiated on capmatinib, resulting in sustained disease stability and a reduction in the lesion size over a three-year follow-up. This case highlights the clinical utility of molecular testing in NSCLC, particularly for identifying MET exon 14 alterations that guide targeted therapy. Capmatinib demonstrated durable disease control and was well tolerated, offering a viable alternative to conventional chemotherapy in an elderly patient with significant comorbidities. These findings support the integration of precision oncology into the management of advanced NSCLC and underscore the potential of MET TKIs to improve outcomes in this high-risk subgroup.

## Introduction

Lung cancer remains the leading cause of cancer-related deaths in both men and women, accounting for 22% of all adult cancer fatalities as of 2021, with a five-year survival rate of only 25%. Non-small cell lung cancer (NSCLC) represents approximately 80% of all lung cancer cases, with adenocarcinoma comprising over 57% of these [1-4]. Early diagnosis significantly improves survival rates; however, lung cancer is often identified at advanced stages when therapeutic options are limited. The five-year survival rate for NSCLC varies substantially by stage, with a rate of 65% for localized disease compared to just 9% for metastatic disease [2].

Metastasis is a complex process, with MET exon 14 skipping mutations and amplifications playing critical roles in the progression of NSCLC. These genetic alterations, present in approximately 3%-4% of cases, are associated with poorer prognoses [5-9]. Notably, there appears to be no strong correlation between MET exon 14 alterations and patient gender, smoking status, ethnicity, or geographical origin [9,10].

Treating elderly patients with advanced NSCLC poses several challenges. Older adults often present with multiple comorbidities, decreased physiological reserve, and higher risk for treatment-related toxicity. Chemotherapy is frequently less well tolerated in this population, and performance status, organ function, and quality of life must be carefully balanced against therapeutic benefits. As such, the availability of targeted therapies with favorable safety profiles, such as a MET tyrosine kinase inhibitor (TKI), offers a significant advantage for this vulnerable group.

As our understanding of cancer biology has advanced, oncology has shifted toward a more personalized approach. Targeted therapies have emerged as a cornerstone in managing molecularly defined NSCLC subtypes. Capmatinib, a selective MET TKI, has recently shown promise as a monotherapy for MET-dysregulated NSCLC [8]. Here, we report the case of an 83-year-old patient with stage 4A metastatic lung adenocarcinoma harboring a MET exon 14 skipping mutation who achieved long-term disease stability following capmatinib therapy.

## Case presentation

**Figure 1:**
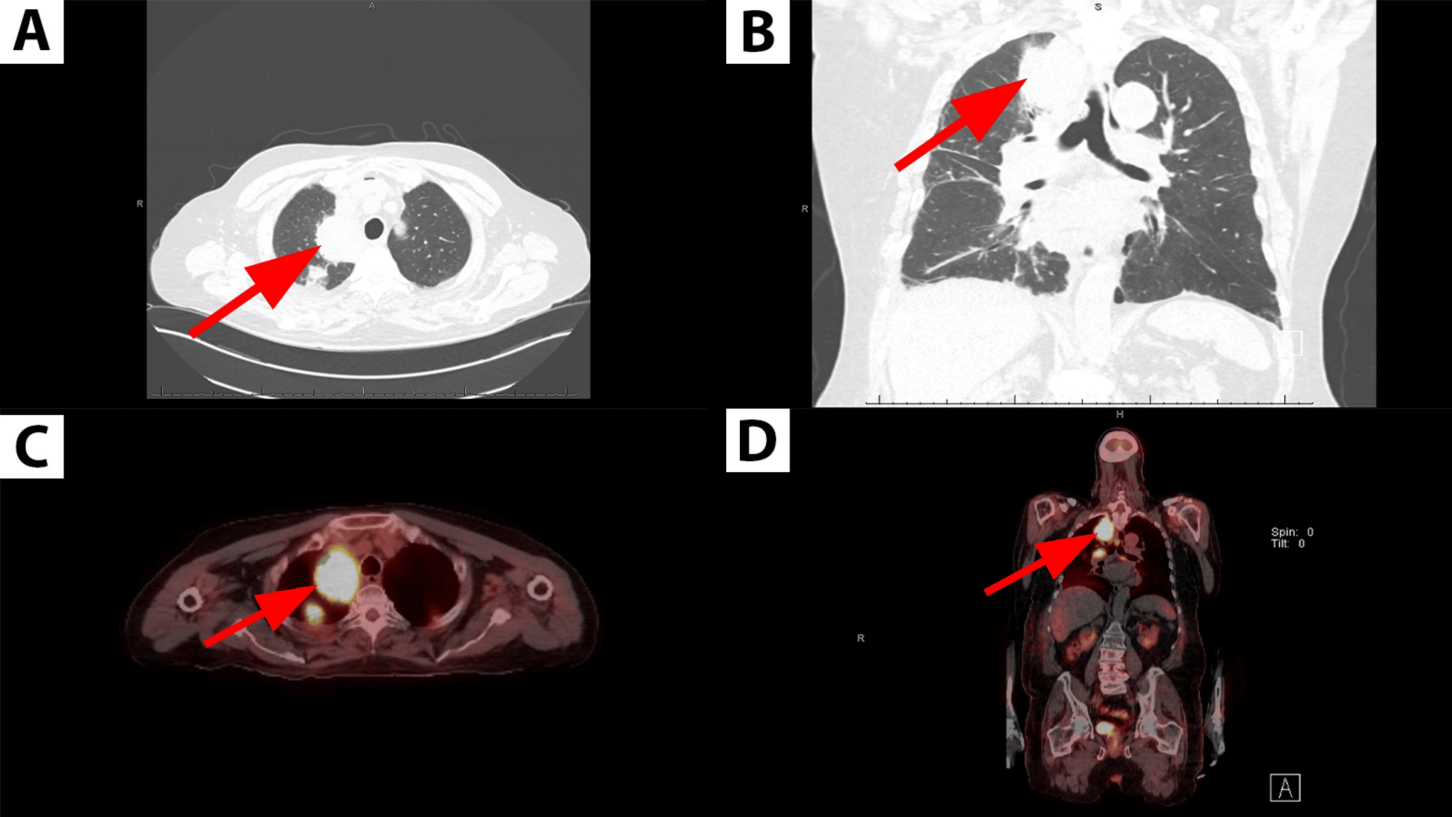
Initial axial and coronal images of chest CT and PET-CT. A. Axial view of chest CT showing a right apical lung mass (red arrow) accompanied by multiple bilateral solid nodules. B. Coronal view of chest CT showing the irregular and heterogeneous right apical mass. C. Axial view of the PET-CT demonstrating hypermetabolic activity of the right apical lung mass (red arrow). D. Coronal view of the PET-CT highlighting hypermetabolic mass and associated nodule.

An 83-year-old former smoker with a history of chronic obstructive pulmonary disease (COPD) presented with acute respiratory failure. Chest computed tomography (CT) revealed a moderately large pericardial effusion with cardiac chamber compression and a large, irregular, heterogeneous right apical lung mass accompanied by multiple bilateral solid nodules (Figures 1A, 1B). Following stabilization and non-diagnostic pericardiocentesis, a subsequent positron emission tomography-computed tomography (PET-CT) scan showed a hypermetabolic lung mass, mediastinal lymphadenopathy, bilateral pulmonary nodules, and pericardial lesions consistent with metastatic disease (Figures 1C, 1D).

**Figure 2:**
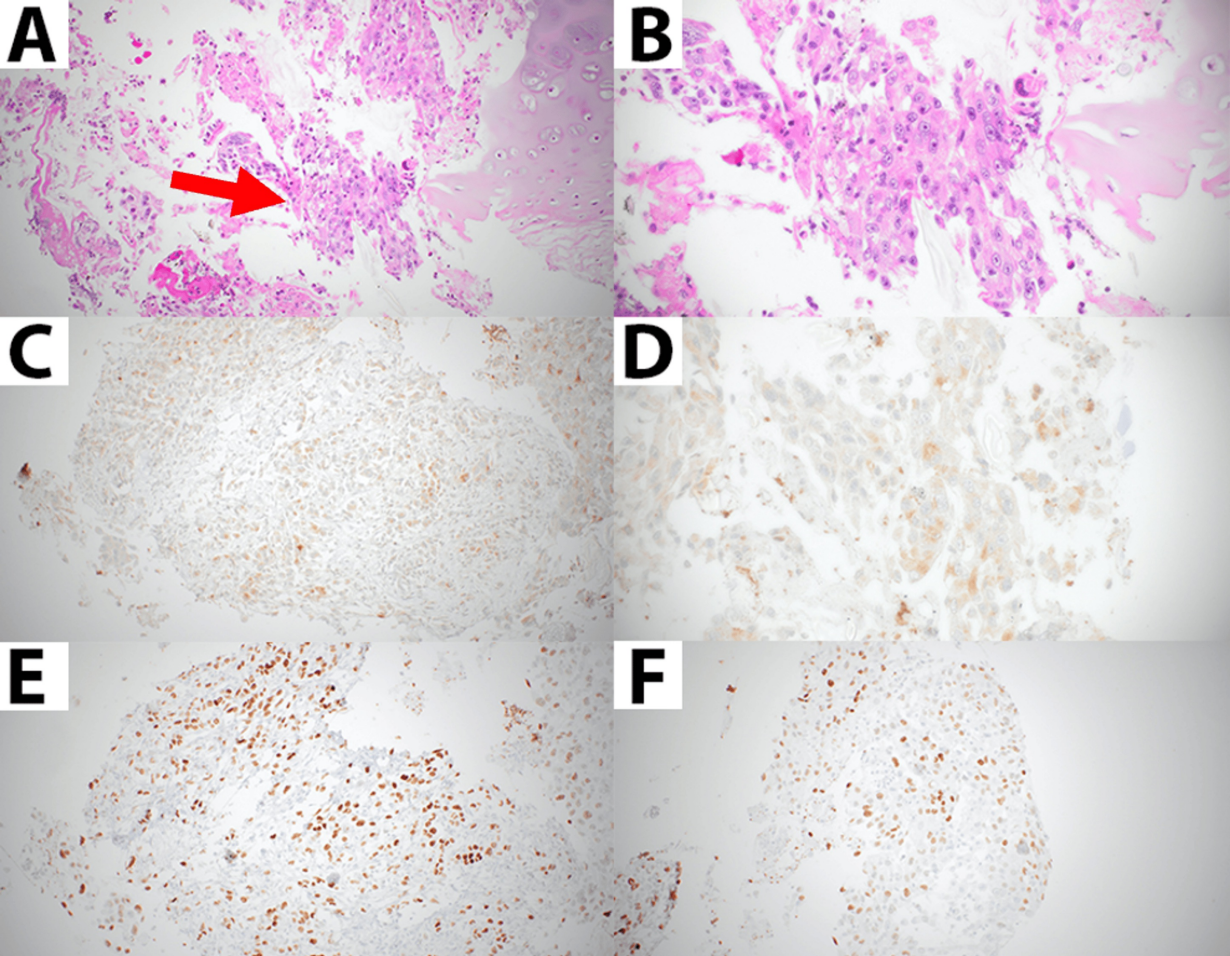
Histological findings of right apical lung mass. A. Hematoxylin and eosin (H&E) immunohistochemical staining showing malignant cells with cytoplasmic vacuolization, prominent nucleoli and irregular nuclei (red arrow)(20x magnification used). B. Magnified image of H&E stain highlighting malignant cells (40x magnification used). C. Immunohistochemical staining positive for Napsin A (20x magnification used). D. Immunohistochemical staining positive for Napsin A (40x magnification used). E. Immunohistochemical staining positive for TTF-1 (20x magnification used). F. Immunohistochemical staining positive for TTF-1 (20x magnification used).

Bronchoscopy and endobronchial ultrasound (EBUS) with biopsy of the right upper lobe apical mass and mediastinal station 4R confirmed the diagnosis of lung adenocarcinoma (Figures 2A, 2B). Immunohistochemical staining was positive for Napsin A and TTF-1 (Figures 2C-2F), and molecular testing showed elevated Programmed Death-Ligand 1 (PD-L1) expression over 90% in tumor cells.

**Figure 3:**
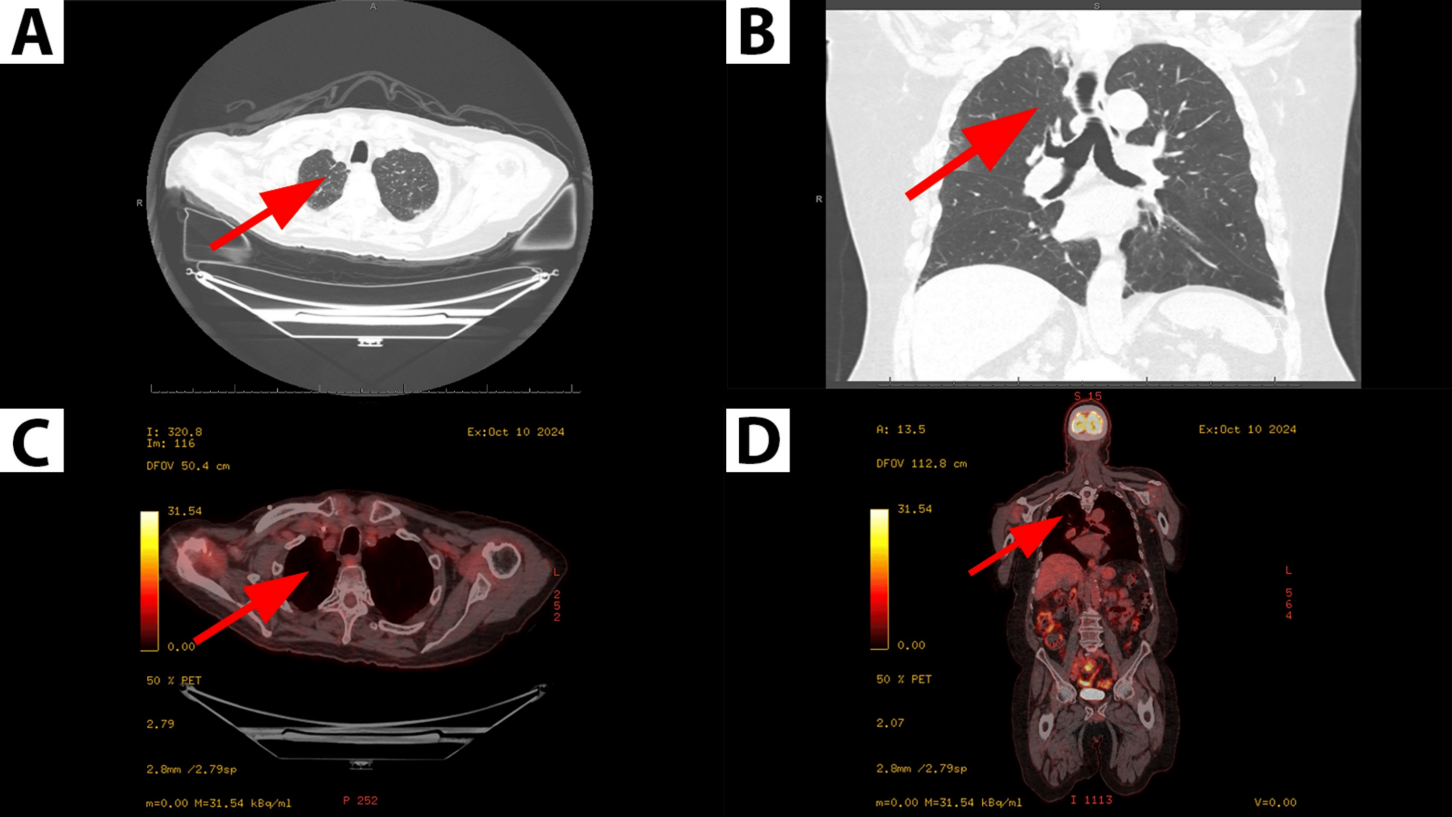
Three-year follow-up axial and coronal images of chest CT and PET-CT. A and B: Axial and coronal views of chest CT showing reduced size of right apical mass (red arrow) and less disease burden. C and D: Axial and coronal views of PET-CT showing reduced hypermetabolic activity (red arrow).

Genomic profiling revealed a MET exon 14 skipping mutation. Based on these findings, the patient was initiated on capmatinib therapy at the standard dose of 400 mg twice daily. He experienced no dose-limiting toxicities during the course of treatment. Mild peripheral edema and transient fatigue were noted in the first six months, managed conservatively without dose reduction. No hospitalizations occurred after treatment initiation. Over a three-year follow-up period, the patient has demonstrated sustained disease stability and a reduction in the size of hypermetabolic lesions as documented on follow-up CT and PET imaging (Figures 3A-3D). He remained functionally independent, with reported improvement in baseline respiratory symptoms due to reduced tumor burden. From a quality-of-life standpoint, the patient reported satisfaction with therapy due to minimal side effects and preserved autonomy.

## Discussion

This case underscores the critical role of molecular profiling in identifying actionable mutations in NSCLC, particularly MET exon 14 skipping alterations. Although these mutations are relatively uncommon, present in approximately 3-4% of NSCLC cases, they are associated with aggressive tumor biology and poorer clinical outcomes [5-9,11]. Capmatinib, a selective MET TKI, has been approved by the FDA for use in patients with MET exon 14-mutated NSCLC based on favorable response rates demonstrated in the GEOMETRY mono-1 trial [11]. However, translating these trial results into real-world practice, especially for elderly patients with significant comorbidities, remains a clinical challenge.

Our patient, aged 83 with a history of COPD, represents a population underrepresented in most clinical trials. Advanced age is a known barrier to aggressive cancer treatment due to decreased physiological reserve, frailty, and polypharmacy. Chemotherapy in elderly patients is often poorly tolerated and can lead to significant morbidity. Even in patients with high PD-L1 expression, immunotherapy has shown limited efficacy in MET-driven NSCLC, and there is growing evidence that MET exon 14 mutations may confer resistance to immune checkpoint blockade [9,10]. Thus, treatment decisions in this demographic must carefully balance efficacy with safety and quality of life.

Targeted therapies have demonstrated significantly improved response rates in patients with MET alterations. A review of 39 studies revealed that first-line treatment with MET TKIs had a median response rate of 50.7%-68.8%, compared to 33.3% for immunotherapy and 23.1%-27.0% for chemotherapy [9,10]. Capmatinib, a selective MET TKI, has demonstrated substantial efficacy in MET exon 14-mutated NSCLC. In the GEOMETRY mono-1 trial, capmatinib showed overall response rates of 68% in treatment-naïve patients and 41% in previously treated patients, with a manageable safety profile [11]. Similarly, tepotinib (another selective MET TKI) demonstrated response rates of 57.3% in treatment-naïve patients and 45.0% in previously treated patients in the VISION trial [12].

This case is particularly noteworthy for the exceptional duration of response: over a three-year period, the patient experienced sustained disease stability and gradual reduction in tumor burden without requiring any dose modifications or experiencing severe adverse events. Peripheral edema and mild fatigue were the only side effects reported, both of which were self-limiting and managed conservatively. Throughout the treatment course, the patient maintained a good performance status, functional independence, and an acceptable quality of life. Outcomes that are especially meaningful in elderly patients with advanced disease.

The safety of MET TKIs has been studied extensively. Most adverse events are manageable and include peripheral edema, nausea, fatigue, and elevated liver enzymes [13]. The durability and tolerability of capmatinib observed in this case highlight its potential utility beyond the controlled environment of clinical trials. While randomized data confirm the drug’s efficacy in the broader population, this report provides valuable real-world evidence of capmatinib’s use in an octogenarian patient with metastatic NSCLC and multiple comorbidities. Furthermore, the case reinforces the importance of molecular testing, even in patients who might initially appear poor candidates for targeted therapy due to age or frailty.

As the landscape of precision oncology continues to evolve, real-world case data remain essential for guiding clinical decision-making, particularly in populations historically excluded from large trials. Ongoing research is exploring mechanisms of acquired resistance to MET inhibitors, potential benefits of combination therapies, and the development of next-generation MET-targeting agents [8,14]. These future directions may further expand treatment options for this challenging subgroup.

## Conclusions

This case highlights the importance of early molecular testing in guiding personalized treatment strategies for NSCLC. It demonstrates how targeted therapy can produce meaningful and durable clinical responses, even in patients with advanced disease and multiple comorbidities. The case also underscores the value of a multidisciplinary diagnostic approach combining imaging, bronchoscopy with EBUS, histopathology, and genomic profiling to facilitate precise therapeutic decision-making.

Identification of actionable alterations, such as MET exon 14 skipping mutations, can significantly impact outcomes, particularly in elderly patients who may not tolerate conventional chemotherapy. Capmatinib, a selective MET TKI, offered a well-tolerated and effective monotherapy option in this case, enabling an 83-year-old patient with stage 4A metastatic lung adenocarcinoma to achieve prolonged disease stability. As research evolves, incorporating routine molecular profiling into NSCLC care pathways will be essential to advancing individualized treatment and improving survival outcomes across diverse patient populations.
